# A Soft Spot for Chemistry–Current Taxonomic and Evolutionary Implications of Sponge Secondary Metabolite Distribution

**DOI:** 10.3390/md19080448

**Published:** 2021-08-04

**Authors:** Adrian Galitz, Yoichi Nakao, Peter J. Schupp, Gert Wörheide, Dirk Erpenbeck

**Affiliations:** 1 Department of Earth and Environmental Sciences, Palaeontology & Geobiology, Ludwig-Maximilians-Universität München, 80333 Munich, Germany; a.galitz@lrz.uni-muenchen.de (A.G.); woerheide@lmu.de (G.W.); 2 Graduate School of Advanced Science and Engineering, Waseda University, Shinjuku-ku, Tokyo 169-8555, Japan; ayocha@waseda.jp; 3 Institute for Chemistry and Biology of the Marine Environment (ICBM), Carl-von-Ossietzky University Oldenburg, 26111 Wilhelmshaven, Germany; peter.schupp@uni-oldenburg.de; 4 Helmholtz Institute for Functional Marine Biodiversity, University of Oldenburg (HIFMB), 26129 Oldenburg, Germany; 5 GeoBio-Center, Ludwig-Maximilians-Universität München, 80333 Munich, Germany; 6 SNSB-Bavarian State Collection of Palaeontology and Geology, 80333 Munich, Germany

**Keywords:** bioactivity, marine sponge, secondary metabolite, natural product evolution, chemotaxonomy

## Abstract

Marine sponges are the most prolific marine sources for discovery of novel bioactive compounds. Sponge secondary metabolites are sought-after for their potential in pharmaceutical applications, and in the past, they were also used as taxonomic markers alongside the difficult and homoplasy-prone sponge morphology for species delineation (chemotaxonomy). The understanding of phylogenetic distribution and distinctiveness of metabolites to sponge lineages is pivotal to reveal pathways and evolution of compound production in sponges. This benefits the discovery rate and yield of bioprospecting for novel marine natural products by identifying lineages with high potential of being new sources of valuable sponge compounds. In this review, we summarize the current biochemical data on sponges and compare the metabolite distribution against a sponge phylogeny. We assess compound specificity to lineages, potential convergences, and suitability as diagnostic phylogenetic markers. Our study finds compound distribution corroborating current (molecular) phylogenetic hypotheses, which include yet unaccepted polyphyly of several demosponge orders and families. Likewise, several compounds and compound groups display a high degree of lineage specificity, which suggests homologous biosynthetic pathways among their taxa, which identifies yet unstudied species of this lineage as promising bioprospecting targets.

## 1. Introduction

Sponges (Phylum Porifera) are among the most successful survivalists in the animal kingdom, originating in the Neoproterozoic (>600 Mio yrs) and with over 9000 species from every aquatic habitat to date, e.g., [1,2,3,4], ranging from tropical reefs, to the deep-sea, arctic waters, and even freshwater bodies see, e.g., [5,6,7,8]. During the Cambrian and the Jurassic, they acted as major reef builders; recent sponge reefs are however not as extensive and can only be found in arctic deep waters on the western Canadian continental shelf, formed by hexactinellid sponge communities [9,10]. In modern coral reefs, sponges fulfil a key role in the ecosystem as nutrient and carbon recyclers, reef consolidators and habitats for micro- and macroorganisms, e.g., [11,12,13].

Their sessile lifestyle constantly exposes sponges to pressure by the presence of spongivorous predators (e.g., turtles, fish, sea stars), by competitors for space (e.g., other sponges, hard and soft corals, bryozoans), and by omnipresent parasites and microorganisms [14,15,16,17,18,19]. Two evolutionary features played an important role for the survival of sponges to the present day, one being an exceptional regenerative potential, allowing them to quickly recover from predatory attacks [20,21,22], the other one being elaborate biochemical defense mechanisms based on bioactive secondary metabolites [23,24]. These complex compounds are either produced by the sponge itself or by one of its numerous microbial symbionts and act as deterrents and self-medication to protect the sponge [25,26,27,28,29].

Since the first discovery of bioactive chemical compounds from sponges in the 1950s by Bergmann and Feeney [30], many other marine organisms (e.g., nudibranchs, algae, bacteria) became known to be producers of bioactive natural products. However, so far, sponges remain by far their most potent sources [31,32], although marine bacteria, especially bacteria and fungi isolated from sponges and other marine invertebrates have gained much attention as sources of novel bioactive compounds [33,34]. While sponge secondary metabolites serve important roles for the defense and survival of sponges, their various effects (e.g., anti-inflammatory, anti-bacterial, anti-viral, anti-platelet, anti-cancer, etc.) also make them attractive for potential pharmaceutical applications [35,36,37]. Despite many of these natural or synthesized compounds being tested in clinical trials, only few drugs are approved by the various regulatory agencies (e.g., US Food and Drug Administration, FDA) and are readily available on the market yet, such as Cytosar, AZT (azidothymidine), or Remdisivir [38,39,40]. With the omnipresent and increasing danger of multiresistant germs and new viral diseases, as well as high interest in new cancer medications, the scientific and commercial interest in new sponge metabolites, and especially their synthetic analogs, is in constantly high demand [41,42,43].

Although the discovery of new marine metabolites and their synthesis for medicinal application are the main priorities of current sponge biochemistry, some of these compounds were once also regarded as potential markers for sponge taxonomy, as substitute or extension of classical morphology-based sponge classification [44,45]. However, it became apparent that these compound-driven chemosystematics could not fulfil the initial expectations, hence partially losing their importance, while at the same time molecular methods underwent quick advancements and a steep gain in popularity [46,47]. However, despite the availability of these molecular methods, most aspects of currently valid sponge taxonomy are still based on morphological characters, often leading to conflicting results and relationships between molecular and morphological phylogenies, e.g., [48,49]. The usage of sponge morphology for classification is prone to error due to paucity of clear-cut discriminating characters coupled with phenotypic plasticity, which likewise impedes correct identification of the metabolite bearing sponge species; see, e.g., [50,51].

In contrast to the relationships among sponges, knowledge on the evolution, interactions, and the production pathways of their secondary metabolites is comparatively scarce, e.g., [52,53]. Getting a better understanding of the relationships among sponge clades with respect to their compound production (and composition) will further bioprospecting and pharmaceutical biotechnology of sponges. Literature based research on sponge compounds, as conducted here, and subsequent compilation of metabolite distribution can be impeded and distorted by a number of obstacles, e.g., [45,54]. The most commonly encountered problems are biased focuses on certain compound groups and/or promising bioactive species, seemingly homologous natural products with non-homologous production pathways, sponge–sponge overgrowth and contamination, and especially insufficient or even misidentified sponge specimens, causing false taxonomic assignments [46]. Most extant sponges live in symbiotic relationships with photosynthetic and heterotrophic bacteria or other microorganisms, thus featuring a pronounced microbiome [55]. Often it is not evident whether the sponge, its symbionts, or a combination of both are responsible for the production of certain metabolites, e.g., [56]. Generally disregarding symbiont-produced bioactive compounds as taxonomically irrelevant would however be a mistake, as part of the microbiome can be highly sponge-specific as well [57].

More than a decade ago, Erpenbeck and van Soest [46] compiled a comprehensive overview of sponge-specific and thus chemotaxonomically relevant metabolites. Although there have been a number of publications reviewing separate sponge compound groups, there has not been a general overview since, e.g., [58,59]. Thus, in the following, we compiled all the recent biochemical publications on sponges and reviews of the last decade in order to aid tracing the taxonomic distribution of compounds based on our current understanding of demosponge phylogeny, which changed considerably in the last couple of years [60].

We believe that the insights we gained here will contribute to the resolution of current and future conflicts in Porifera taxonomy but particularly facilitate the discovery rate and taxonomic accuracy of sponge bioprospecting.

## 2. Methods

**Figure 1:**
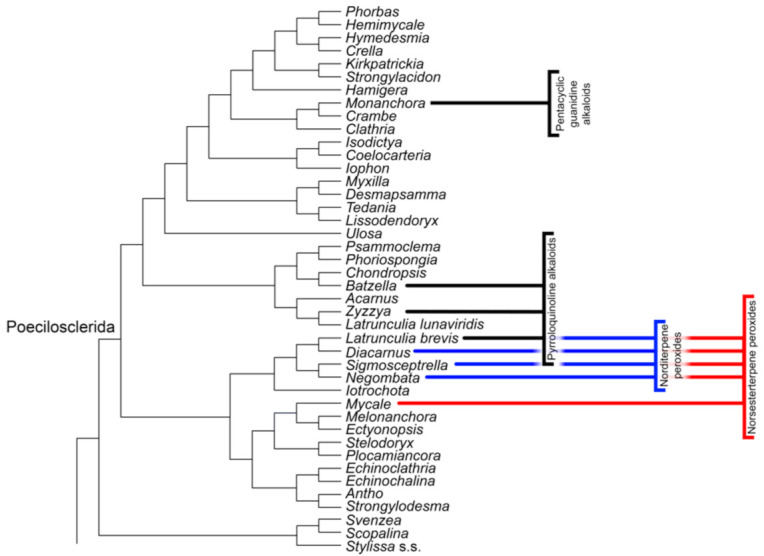

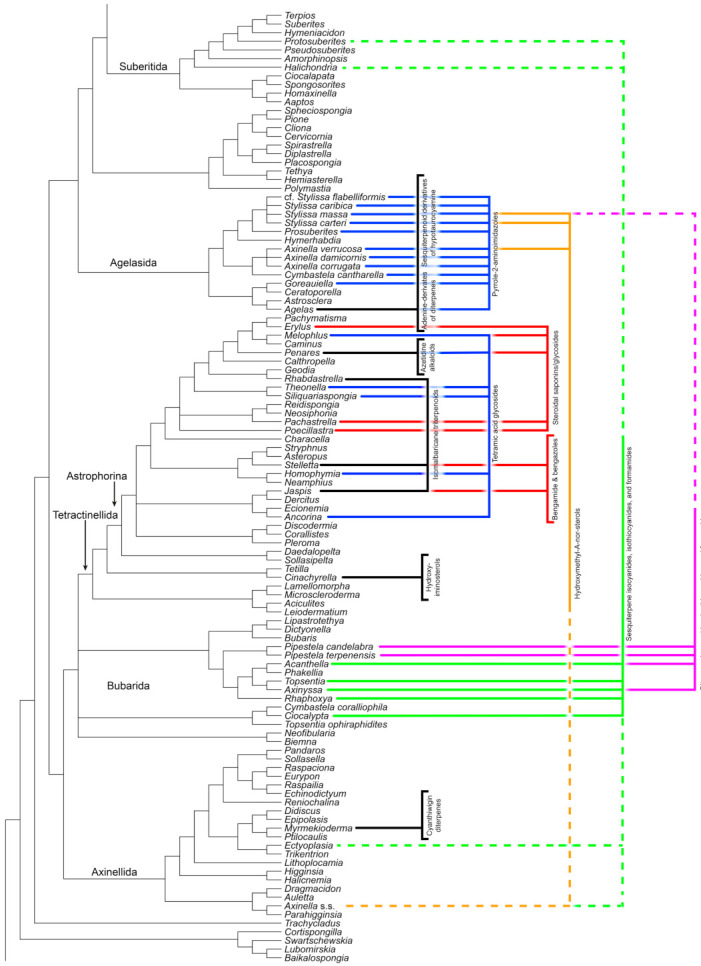

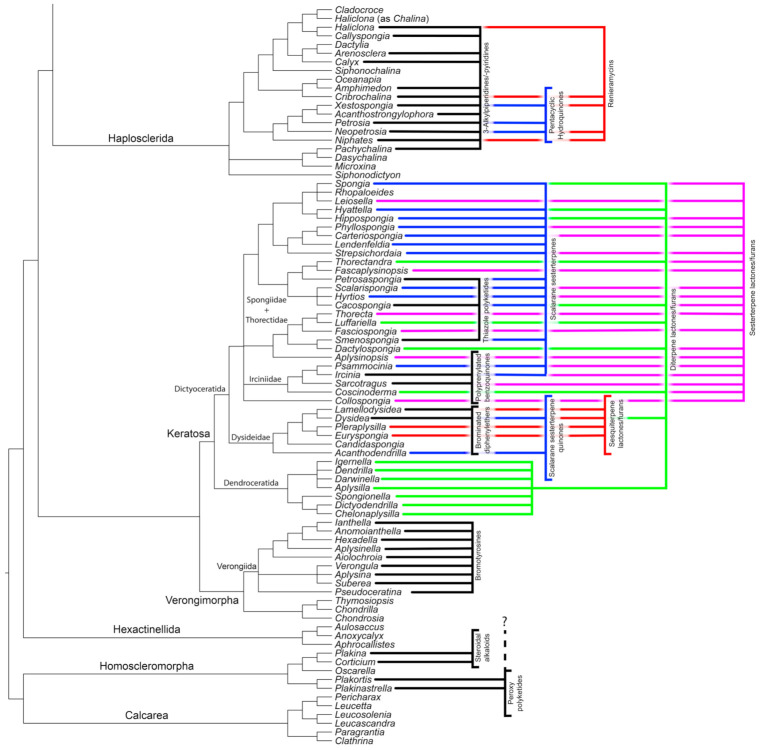
Phylogenetic distribution of bioactive sponge compounds. Taxa were chosen from a comprehensive list of metabolite-bearing sponge species, independent of their taxon specificity, and were supplemented with further taxa from the respective molecular tree sources where applicable. Colors do not depict relatedness of compounds and were solely chosen for better contrast between different compound classes. Dashed lines indicate reports of compounds suggested for verification. Genus and species names have been adopted from the respective source publications. Particularly for taxa that still await revision, higher-level classification (as given on the branches) might be in conflict with the current reference (World Porifera Database). See text for details.

The evaluation of the current status and distribution of secondary metabolites from sponges is based on the approach of Erpenbeck and van Soest [46] (obtained from the MarinLit database and data from van Soest and Braekman [45]) and subsequently expanded upon it with data from the annually released review of Marine Natural Products, e.g., from 2005 to 2017 [31,32], as well as additional singular publications. Metabolites were separated into major chemical compound categories and plotted against a combined molecular phylogenetic consensus tree for all sponge classes based on some of the latest studies available for the different sponge taxa (Figure 1), e.g., [61,62,63,64,65,66,67]. Compound groups commonly known for production by microorganisms were generally disregarded due to frequent symbioses with a plethora of different sponge species, unless host specificity could be verified with sufficient reliability. Validity and status of the sponge taxa as named in the individual publications was checked against the World Porifera Database [4] and the Systema Porifera [68].

## 3. Specificity and Phylogenetic Relevance of Sponge Compounds

### 3.1. General

The updated and supplemented dataset created by Erpenbeck and van Soest [46] could be further expanded by over 1100 new metabolite reports from over 160 genera, finally comprising 80 compound classes from over 850 different sponge species. Based on this data, 30 characteristic, and potentially lineage-specific, metabolite classes (henceforth also referred to as “markers”) could be identified, spanning 11 sponge orders in Demospongiae and Homoscleromorpha (see Table 1 and Figure 1). No conclusive evidence of new markers for Calcarea or Hexactinellida could be found since 2007.

### 3.2. Demospongiae

Extant demosponges are currently divided into three subclasses: Heteroscleromorpha comprises species (mostly) possessing siliceous spicules, Verongimorpha and Keratosa with (mostly) aspiculous species [60].

#### 3.2.1. Heteroscleromorpha

##### Poecilosclerida

Poecilosclerida constitute the largest demosponge order in terms of taxon [69] and supported distinct from other orders due to the joint possession of characteristic skeletal elements “chelae” and its derivatives, [70] and molecular phylogenetic reconstructions [67].

**Figure 2:**
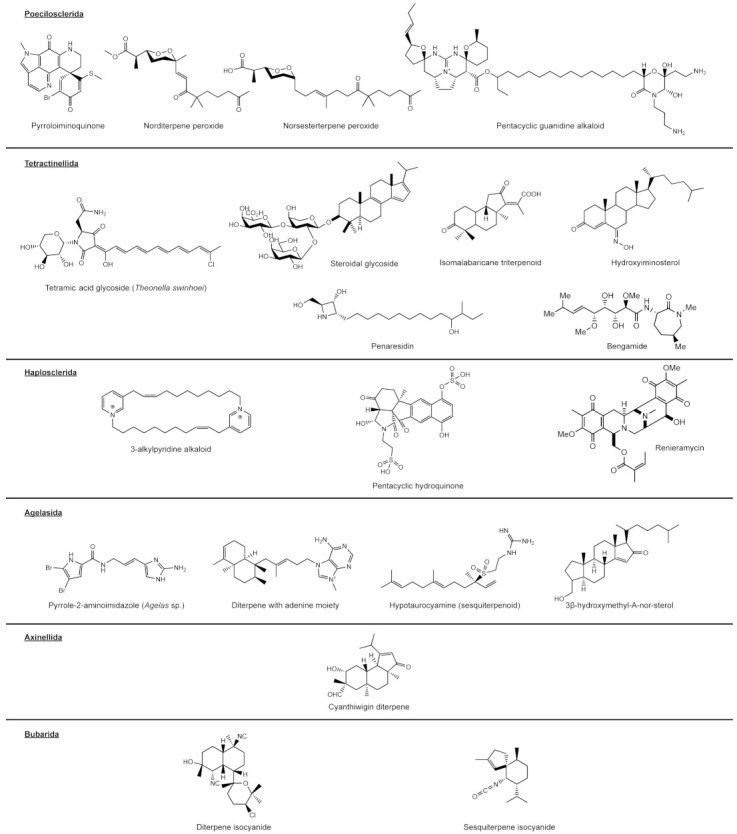
Selection of exemplary sponge-derived secondary metabolites with potential for taxon specificity. The value and validity of the investigated compound groups are discussed in their respective sections of the text.

Norditerpene peroxides pose a potential marker exclusive to the family of Podospongiidae within Poecilosclerida, e.g., [71,72] (Figure 2). The related norsesterterpene peroxides are also found outside of this family in several specimens of *Mycale* and *Latrunculia*, as discussed in van Soest and Braekman [45]. This would expand the range of norsesterterpenes as marker for higher poecilosclerid lineages, misidentifications of podospongiid sponges for the stated genera can however not unequivocally be ruled out (Figure 1).

Pyrroloquinoline, or pyrroloiminoquinone alkaloids, are frequently found in members of the molecularly closely related families of Acarnidae (*Zyzzya*) and Chrondropsidae (*Batzella*), making them a well-supported marker for these clades [73]. The detection of pyrroloiminoquinone alkaloids in *Latrunculia brevis* [74] and other species of this genus e.g., [75,76,77] lends further support to this metabolite group being a reliable indicator for Poecilosclerida, while the phylogenetic position of *L. lunaviridis* close to Acarnidae (Figure 1) indicates the general need of thorough, interdisciplinary investigation of the source material (see [78] for a good example on latrunculids), as species of *Latrunculia* are generally well described with clear morphological relationships [70]. 

Pentacyclic guanidine alkaloids might represent a new marker exclusive for the Crambeidae genus *Monanchora,* e.g., [79]. While polycyclic and especially tricyclic guanidine alkaloids can also be frequently found in Poecilosclerida, they seem to not be restricted to this order and are also found in Axinellida, Biemnida, and Bubarida, e.g., [80,81].

##### Tetractinellida

Tetractinellida constitutes a distinct demosponge order as reflected in characteristic morphology, e.g., [60], supported molecular phylogenies, e.g., [82,83], and also in its distinct biochemical compounds.

Tetramic acid glycosides are well known compounds produced by various tetractinellid families (Ancorinidae, Geodiidae, Neopeltidae, Theonellidae) among the suborder Astrophorina (see Figure 1), which were suggested as distinct markers for these families [46]. Due to the documented production of this compound class by fungi, the authors did however note its ambiguous specificity for sponges, e.g., [84]. Tetramic acids without glycosidic moiety can also be found in other sponge taxa, as well as in sponge-derived fungi, making this glycosidic moiety specific for Tetractinellida [85,86].

Steroidal saponins and glycosides, besides being commonly found in many Echinodermata [87], also have been reported in sponges. Since the compounds have been mainly reported from specimens of the suborder Astrophorina, they were initially considered as evolutionary characteristics for this clade but were disregarded due to studies from non-tetractinellid genera [46,88]. As Ivanchina et al. [89] stated, there are, however, major structural differences among glycosides in sponges, with sparse reports of these metabolites outside of Astrophorina possibly being rare homologs. Although all recent metabolites reports refer to Astrophorina, the aforementioned outliers (e.g., *Pandaros*, *Niphates*, *Ectyoplasia*) should not be disregarded, hence rendering the specificity of these metabolites questionable [90,91,92], especially when taking into account their occurrence in other invertebrates [87].

Likewise, triterpenoid saponins, which also commonly occur in echinoderms, are frequently found in the suborder of Astrophorina, especially among members of the family Geodiidae, e.g., [93,94]. Their concentrated occurrence made these compounds additional potential Astrophorina markers; however, multiple reports from various unrelated taxa diminish their suitability greatly, e.g., [95,96].

While isomalabaricane triterpenoids were considered to be robust markers for the astrophorid *Rhabdastrella* [46], and previous findings in the related genera of *Jaspis*, *Geodia* and *Stelletta* had been ruled out as misidentifications [45], several recent reports from the latter taxa now contradict this theory, with at least *Jaspis* being verified in two instances [97,98]. Based on these reports, isomalabaricane triterpenoids at least remain a marker for the suborder Astrophorina.

Metabolites from the classes bengamides, bengazoles, and their derivatives are known from few Astrophorina genera, with *Jaspis* being the most prominent, e.g., [99,100]. Van Soest and Braekman [45] already suggested these compounds as being exclusive to this suborder, but due to their resemblance to bacterial fatty acids, they did not commit to this decision. A more recent review by White et al. [101], however, supports the assessment of specificity and even implies that most other Astrophorina sponges from older studies actually were misidentified *Jaspis* specimens, with multiple *Jaspis* and *Stelletta* species formerly being assigned to the genus *Dorypleres*. A combination of these findings with the occurrence of bengamides in *Stelletta* [102] makes bengamides, bengazoles, and their derivatives specific for the family Ancorinidae, but also highlights the complex taxonomic situation between *Jaspis* and *Stelletta*, prompting for a possible revision of these genera.

Naturally acetylated glycolipids are rarely occurring compounds in sponges, mainly reported from the geodiid taxa *Caminus*, *Pachymatisma* and *Erylus*, making them a potentially distinct marker for Geodiidae [103]. Contradicting this assessment are however discoveries in the Axinellida genera *Trikentrion* and *Myrmekioderma,* e.g., [104]. Despite the large variety, lack of specificity, and often symbiotic (co-)production of lipids in sponges, these rare acetylated metabolites appear to be largely confined to species of tetractinellid and axinellid sponges (see Figure 1). Furthermore, according to Wjonar and Northcote [105], these comparatively uncommon compounds might often go unnoticed due to the frequent use of acetylation for the isolation of glycolipids [106]. Since there are no obvious structural differences between the tetractinellid and axinellid glycolipids, we assume their analogous origin in both orders.

Within Tetractinellida, the spirophorid genus *Cinachyrella* is characterized by the presence of certain oxime containing sterols, the hydroxyiminosterols. These were already dubbed potential markers by Erpenbeck and van Soest [46], which gains further support by recent findings [107].

With no recent reports of azetidine alkaloids, like penaresidin and penazetidine, this group of metabolites retains its status as a highly likely marker for the ancorinid genus *Penares* [45,46].

##### Haplosclerida

Molecularly, the distinctiveness of the order Haplosclerida is reflected in particular structural ribosomal features [108] and subsequent molecular phylogenies, e.g., [109], although internal phylogeny of this order remains yet to be unraveled, e.g., [110] and subsequent publications of the McCormack group.

3-alkylpyridine and 3-alkylpiperidine alkaloids are compounds typically found across all haplosclerid families but the Phloeodictyidae and thus were considered as taxon-specific metabolite class, although reports from other sponge taxa undermined this assumption, e.g., [111,112]. With the overwhelming majority of older and more recent reports being almost exclusively limited to Haplosclerida, correct reports from other orders seem increasingly unlikely, although few studies on the Suberitida family Halichondriidae claim to have found compounds identical to those from *Haliclona,* e.g., [113]. Without inspection of the original sponge material, misidentifications or sponge–sponge contaminations in these rare cases cannot be completely ruled out. The commonness of these alkaloids in Haplosclerida, however, strongly supports the validity of 3-alkylpyridine and 3-alkylpiperidine alkaloids as a specific marker.

More specific and less controversial markers are pentacyclic hydroquinones found in Petrosiidae sponges. Non-terpenoid quinones are comparably rare compounds found among sponges, especially the pentacyclic, as well as in few cases hexacyclic, variants found in specimens of *Petrosia* and *Neopetrosia,* e.g., [114,115].

Renieramycin-type metabolites from the family of tetrahydroisoquinolines are frequently found in different haplosclerid sponges, e.g., *Haliclona*, *Xestospongia*, and *Cribrochalina* [116]. Their taxonomic specificity was however doubted by van Soest and Braekman [45] and Erpenbeck and van Soest [46] due to the possibility of bacterial origin. This assessment is corroborated by recent findings of Tianero et al. [117] of highly specific bacterial symbionts in a species of *Haliclona*, also unravelling the biosynthetic pathways and host-symbiont relationships on a cellular level. These results would imply similar mechanisms for related sponges and would support renieramycins as characteristic metabolites for Haplosclerida.

Straight-chain polyacetylenes are compounds previously considered to be taxonomically distinct to the order Haplosclerida, which was subsequently restricted to acetylenes with bromine (*Xestospongia*) or hydroxylic (*Petrosia*) moieties [45,46]. While recent reports from Haplosclerida still vastly outnumber any other sponge taxa, further polyacetylenes from non-haplosclerid sponges have been discovered as well, some of which also seem to bear brominated or hydroxylated side chains [118]. Although the majority of sponge-derived polyacetylenes have long chain lengths, there also are C_15_ and short-chain (less than C_15_) acetylenic compounds, which appear more specific for Haplosclerida. However, some C_15_ polyacetylenes have been found both in sponges and algae, making the exact origin of these metabolites more ambiguous [119]. Consequently, a thorough investigation of polyacetylenic metabolites found within and outside of Haplosclerida is necessary to evaluate the taxonomic specificity of straight-chain polyacetylenes.

##### On Agelasida, Axinellida, Bubarida, and Suberitida

The classification of the genera from the current orders Agelasida, Axinellida, Bubarida, and Suberitida experienced a major turmoil in the last couple of years when molecular data revealed eminent shortcomings in the traditional (morphological) classification due to the lack of unambiguous morphological discriminatory apomorphies (see Erpenbeck, Hall et al. [62] and Wörheide et al. [47] for an overview). Still, the position of many taxa in the current classification [60] awaits robust molecular support, while several genera have subsequently been recovered as polyphyletic, e.g., [63,64]. The uncertain classification complicates estimation of the taxonomic range of metabolites from the literature alone.

##### Agelasida

Pyrrole-2-aminoimidazoles (P-2-AI), also called bromopyrroles, pyrrole-imidazole alkaloids, or pyrrole-2-carboxylic acid derivatives, have been proposed multiple times as chemotaxonomic markers for Agelasida, e.g., [44,46,58,120]. Since these metabolites are also commonly found in certain specimens classified as *Axinella* spp. and *Stylissa* spp., Braekman et al. [120] suggested a closer relationship of these genera to Agelasida. Indeed, molecular data have revealed *Axinella* as polyphyletic, e.g., [48,63] with the P-2-AI producing species *A. corrugata*, *A. damicornis,* and *A. verrucosa* being distant from *Axinella* sensu stricto (that include the type species *A. polypoides*) [63] and in close relationship to Agelasida, e.g., [48]. Similarly, the genus *Stylissa* is found as nonmonophyletic, with the P-2-AI producing species (incl. *S. carteri* and *S. massa*) forming a clade with Agelasida [49,121] and distant to the nominal type species *S. flabelliformis* (Order Scopalinida) [122]. Subsequently, Morrow et al. [65] classified some of the divergent *Axinella* and *Stylissa* species into a new family Hymerhabdiidae inside a re-defined order Agalasida. The production of P-2-AI in *Cymbastela cantharella* [123] and *Prosuberites laughlini* [124] is subsequently reflected by their molecular phylogenetic position in this clade, e.g., [63,64,65]. This Agelasida sensu Morrow et al. [65] clade is further corroborated by additional recent biochemical reports of P-2-AIs, e.g., [125,126,127,128], as well as molecular phylogenetic studies [67].

Braekman et al. [120] identified and suggested special diterpenes with an adenine moiety, including hypotaurocyamine, as potential apomorphic character for the genus *Agelas*, with numerous recent reports of adenine derivatives of diterpenes from this genus, e.g., [129,130]. These compounds appear characteristic and apomorphic for *Agelas*.

Although not as common as the diterpenoid variants, sesquiterpenoid derivatives of hypotaurocyamine can also be found among Agelasida. Since no new reports contradict the initial assessment of this class of metabolites being specific to the genus *Agelas*, its status as a valid marker persists [120,131].

3β-Hydroxymethyl-A-nor-sterols were previously regarded as potential markers for Axinellidae (Erpenbeck and van Soest [46]). In our current understanding of demosponge phylogeny, hydroxymethyl-A-nor-sterols now appear restricted to Hymerhabdiidae. Besides the records mentioned and discussed in Erpenbeck and van Soest [132] and Erpenbeck and van Soest [46] and new reports solely for “*Axinella*” (=*Stylissa*) *carteri* [133,134]. For reports from *A. polypoides* [135] and *Phakellia* (=*Axinella*) *aruensis* [136], a taxonomic reanalysis is advisable in the light of *Axinella* polyphyly.

##### Axinellida

Cyanthiwigin-type 7-6-5 tricyclic diterpenes of the cyathane family are compounds exclusive to the axinellid genus *Myrmekioderma*. Previous reports of these metabolites from *Higginsia* actually belong to the nigernin-type within the cyathanes [46,137]. Another sponge frequently discussed as cyanthiwigin-containing is “*Epipolasis reiswigi*” [138], which, however, has been synonymized with *Myrmekioderma gyroderma*, hence corroborating cyanthiwigin-type 7-6-5 tricyclic diterpenes as marker unique to *Myrmekioderma*.

##### Bubarida and Suberitida “Incertae Sedis”

Bubarida is a recently erected order consisting of primarily suberitid and axinellid taxa [60] that were molecularly found distant to currently accepted Suberitida or Axinellida, e.g., [62,67]. Polyphyly of several species and the lack of unambiguous molecular data from type species currently hamper genus delimitations, e.g., [48,64].

Terpene isocyanides, isothiocyanides, and formamides often occur together in sponges [139], and hence, we regard them as a single marker. Substituted diterpene variants (diterpene isocyanides) are mainly found in sponges of the order Bubarida, including taxa formerly classified as Axinellida, making them a potential evolutionary apomorphy for this order (see Figure 1). Within the diterpenoid compounds, the class of kalihinanes is only present in sponges of the genus *Acanthella* see review of [140]. The class of amphilectanes, despite being mainly reported from bubarid genera, e.g., [141,142], has been described from taxa outside of this order as well, e.g., *Ectyoplasia ferox* as *Hymeniacidon amphilecta* in [143], *Hymeniacidon* sp. [144], *Halichondria* sp. [145], *Haliclona* sp. as *Adocia* sp., [146], *Svenzea flava,* e.g., [147] *Stylissa massa* as *Ciocalapata* sp., [148], and *Cribrochalina* sp. [149]. Several of these species lack discrete distinguishing morphological characters, and therefore, a taxonomic revision of the material is strongly suggested.

Compared to the diterpenes, the larger group of marine isonitriles and related compounds contain a sesquiterpenoid backbone and are subdivided into nine classes. Similarly to the diterpene variants, these compounds are predominantly found in Bubarida, former members of Suberitida, and closely related species, e.g., [150,151] (see Figure 1), while several have also been described from unrelated taxa. These outliers comprise isonitriloids of the classes axanes, eudesmanes, aromadendranes, and epimaalianes, being reported from *Axinella cannabina,* e.g., [152], while unspecified *Halichondria* sp. were found to contain sesquiterpenes with eudesmane, cadinane, spiroaxane, and bisabolene backbones, e.g., [153]. Further sesquiterpenoids were found in *Ciocalypta* sp. Pupukeane-class; as *Hymeniacidon* sp., [154], *Halichondria panicea* Cadinane-class; [155], *Halichondria* cf. *lendenfeldi* Bisabolene-class; [156], *Phycopsis* sp. Bisabolene-class; [157], and *Theonella* cf. *swinhoei* Bisabolene-class; [158], with this being the only ever report from a sponge not part of the former order of Halichondrida. However, there should be some caution in regards to the assignment of certain sesquiterpenes as phylogenetic markers, since there are also reports of several cadinane sesquiterpenes, the trichodermaloids, produced by the symbiotic fungus *Trichoderma* sp. SM16 isolated from the sponge *Dysidea* sp. [159]. Therefore, it is not unlikely that in some cases associated microorganisms are the actual producers of the detected sesquiterpenes. As discussed in the respective chapter of Erpenbeck and van Soest [132], similarities in the skeletal morphology of former axinellid and halichondrid sponges to each other, as well as to some haplosclerid taxa (e.g., Niphatidae), suggest frequent misidentifications among taxa. Frequently missing identification on species level, as well as geographical occurrences distant from the type locality, e.g., *H. panicea*; [155] further add to this. The current data strongly support both isonitriloid sesquiterpenes and diterpenes as markers for Bubarida, until the mentioned uncertainties are clarified.

Carbonimidic dichlorides, or dichloroimines, constitute a rare class of isonitriloid sesquiterpenoids with both nitrogen and carbon moieties known from formerly halichondrid sponges of the genera *Axinyssa,* e.g., [160,161], *Stylissa massa* [162], and *Ulosa spongia* [163]. Erpenbeck and van Soest [46,132] suggested this compound group as a potential marker for Halichondrida, but the polyphyly of this order (see Figure 1), as well as the reassignment of the aforementioned genera to different orders, now contradict their initial assessment. Aaptamine alkaloids were previously considered as metabolites specific for the family Suberitidae; however, this has been disregarded due to multiple reports from sponges of the orders Haplosclerida and Dictyoceratida [45,46]. Nevertheless, all recent reports are restricted to sponges of the suberitid genus *Aaptos,* e.g., [164,165,166].

Díaz-Marrero et al. [167] suggested suberitane sesterterpenoids as found in *Suberites caminatus* as taxon-specific metabolite for Suberitida, which is contradicted by recent findings of Solanki et al. [168] from the poecilosclerid genus *Phorbas*. Several other related compounds with an “alotane” carbon skeleton as precursor have been reported from Poecilosclerida and Suberitida, implying the possibility of a closer biochemical relationship between these clades [169].

#### 3.2.2. Verongimorpha and Keratosa

Most verongimorph and keratose sponges can be morphologically distinguished from the taxonomically larger group of heteroscleromorph sponges in their inability to produce siliceous skeletal elements of macroscopic scale, although there are exceptions like the aspicular haplosclerid *Dactylia* [170]. While all Keratosa possess some sort of skeleton consisting of spongin fibres, Verongimorpha can either have similar structural elements, microscleric skeletons (*Chondrilla*), or no type of skeleton at all [68].

##### Verongiida (Verongimorpha)

Bromotyrosines were disregarded as a marker for Verongiida in Erpenbeck and van Soest’s [46] review due to sporadic reports from other orders (Poecilosclerida, Agelasida, Tetractinellida, Haplosclerida, Dictyoceratida). However, all recent reports have been restricted exclusively to verongiid taxa, e.g., [171,172]. These conflicting reports displayed structural homologies to the bromotyrosines found in Verongiida but were not sufficiently checked for misidentifications and sponge-sponge contaminations [173]. Based on the new data, bromotyrosines can be regarded as phylogenetic markers for Verongiida. Nevertheless, a secondary loss of bromotyrosine production has recently been documented: Genus *Narrabeena* was classified outside Verongiida due to the absence of bromotyrosines [174], but molecular holotype data confirm the verongiid nature of this genus, indicating secondary losses of bromotyrosine production in Verongiida [175].

#### 3.2.3. Keratosa

The defining morphological differences between the two Keratosa orders Dendroceratida and Dictyoceratida are the eponymous dendritic fiber skeletons, present only in dendroceratid sponges, and their higher tissue-to-fiber ratio, making them softer and more delicate in comparison to the resilient or even hard species of Dictyoceratida [176,177].

Within Dictyoceratida, the family Dysideidae is molecularly distinct and can be morphologically distinguished from the other three families by their choanocyte chamber type [61,178]. Likewise, Irciniidae can be differentiated from thorectid and spongiid sponges by molecular data and the presence of collagenous fibres in the mesohyl [179]. Thorectidae and Spongiidae, however, cannot be recovered as monophyletic [61,178]. These patterns are also apparent when considering the biochemical data.

**Figure 3:**
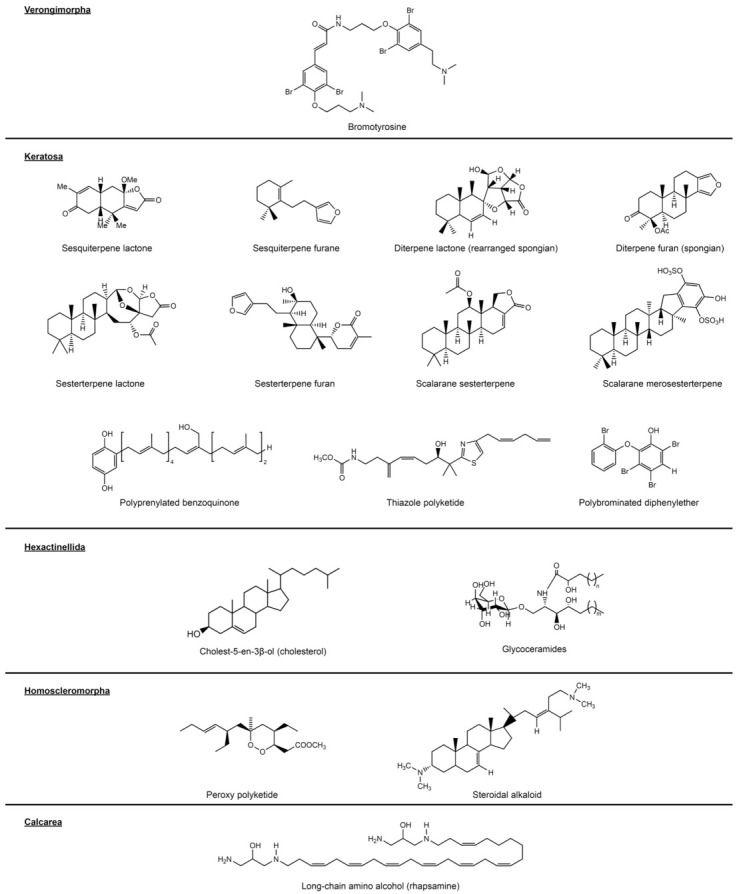
Further exemplary sponge-derived secondary metabolites with potential for taxon specificity. The value and validity of the investigated compound groups is discussed in their respective sections of the text.

Terpene lactones and furans are compounds only found in the keratose orders of Dictyoceratida and Dendroceratida (Figure 3). In many cases, these two types of compounds are found simultaneously, and therefore, lactones and furans will be treated as a singular marker for their respective terpene classes.

Diterpene lactones and furans, including spongiane diterpenes, are the only lactonoid and furanoid metabolites found in both Dictyoceratida and Dendroceratida. While they are inconsistently present in several genera of Dictyoceratida (e.g., *Hippospongia*, *Spongia*, *Luffariella*), especially in the family Spongiidae, they are mainly found in all investigated specimens of Dendroceratida, e.g., [180,181].

Sesquiterpene lactones and furans are mostly restricted to the family Dysideidae with various recent reports strongly supporting this marker’s validity, although singular conflicting reports outside of this clade (e.g., Dendroceratida, Axinellida) remain to be investigated for possible misidentifications or other inconsistencies, e.g., [182,183].

Sesterterpene lactones and furans, on the other hand, are mainly known from the dictyoceratid families of Thorectidae, Spongiidae, and Irciniidae. Although few studies have reported these compounds in dendroceratid genera [184,185,186,187], the increased presence of these metabolites in the aforementioned families makes these findings more likely to be misidentifications, which can be a common issue among the morphologically often hard to distinguish Keratosa sponges [61].

Scalarane merosesterterpenes, or sesterterpene hydroquinones, are rare metabolites only found in *Dysidea* (Dysideidae) [188,189] and more recently in *Acanthodendrilla* (classified as Dendroceratida: Dictyodendrillidae). This supports molecular data that recover *Acanthodendrilla* type material among the Dysideidae [61].

Naturally occurring polybrominated diphenyl ethers are rare and in sponges can only be found in the family Dysideidae, produced by its bacterial symbionts, e.g., [190]. Compounds with microbial origin should generally be considered with caution, due to uncertain host specificity, as well as complex metabolite production pathways and host-symbiont interactions [46,191]. In this regard, polybrominated diphenyl ethers represent a unique case, as both their biosynthetic pathways and cyanobacterial origin could be shown, while still being host-specific to sponges of Dysideidae [192].

Scalarane-type sesterterpenes are limited to the families of Thorectidae, Spongiidae, and Irciniidae within Dictyoceratida, although their distribution is more biased towards specific clades within these complex groups instead of being more evenly distributed like the terpene lactones and furans (see Figure 1) [193].

Polyprenylated benzo- and hydroquinones, despite also being known from the brown algae *Taonia atomaria* [194], are possible markers specific for Irciniidae, with several recent reports from the genera *Ircinia* and *Sarcotragus,* e.g., [194,195].

Similarly rare and specific are thiazole polyketides, currently limited to the genera *Cacospongia*, *Petrosaspongia*, and *Smenospongia* within Thorectidae, e.g., [196,197,198].

### 3.3. Hexactinellida

Glass sponges (Class Hexactinellida) are among the least studied sponge taxa, even more so in terms of biochemistry, owing to their mostly deep-sea habitats and thus comparative scarcity of animal material, as well as the generally low amounts of tissue [45,199].

Blumenberg et al. [200] found a number of “simplistic” sterols (cholesterol and derivatives), which were lacking certain modifications of rings and side-chains of sterols typically found in Demospongiae, thus making them specific for Hexactinellida. While the simplicity of these molecules allows for delimitation from other sponge classes, the missing specificity also makes them unsuitable for use on intraclass levels.

Another lipidoid metabolite exclusive to Hexactinellida was identified by Núñez-Pons et al. [201], who found glycosphingolipids with a specific composition of ceramides, called glycoceramides, which appear to be only present in glass sponges. 

The biochemistry of Hexactinellida, especially their biosynthetic pathways and evolutionary history, still remain largely unknown. They appear to be mostly independent from the other sponge classes, although lipid composition and microbiome put them into a closer relationship to Demospongiae [202].

### 3.4. Homoscleromorpha

The unique feature differentiating sponges of the class Homoscleromorpha from the other sponge classes is the possession of a true basement membrane of collagen IV, typically found in all Metazoa except sponges [203,204]. They constitute the sister group to Calcarea. Like Demospongiae and Hexactinellida, they are able to produce siliceous spicules, however with distinct differences in the biosynthesis [205].

Homoscleromorph sponges are known producers of compounds from the classes of steroidal alkaloids and peroxy-polyketides. Steroidal alkaloids were acknowledged as Homoscleromorpha diagnostic by van Soest and Braekman [45], which is now supported by recent studies, e.g., [206]. Peroxy-polyketides were disregarded as markers by Erpenbeck and van Soest [46] due to multiple reports from other sponge taxa. Despite some recent studies claiming to have found polyketide peroxide metabolites in single taxa such as *Agelas* and *Hippospongia*, the majority of reports originate from homoscleromorph sponges [207], making them potential markers for Homoscleromorpha. Sponge-sponge associations of Homoscleromorpha might constitute a further source for misidentified compound origin (e.g., *Plakortis* and *Agelas*; see [208]).

### 3.5. Calcarea

Similarly to Hexactinellida, reports of new secondary metabolites from calcareous sponges are scarce, due to lacking research focus and unprofitable perspectives. Consequently, there is hardly evidence for any kind of biochemical synapomorphies.

The only exceptions to this are C_27_ to C_29_Δ^5,7,22^ sterols and C_27_ to C_29_Δ^5,7,9(11),22^ sterols found in Calcarea, which were identified by Hagemann et al. [209]. They emphasize that these steroids are different from hexactinellid sterols, while sharing structural similarity with demospongian sterols, making them unsuitable for the resolution of intraclass relationships.

A further calcarean marker, as previously reviewed in Erpenbeck and van Soest [46], is amino alcohols over C_29_ chain lengths for the families Clathrinidae and Leucettidae (both Clathrinida).

## 4. The Legacy of Chemosystematics–Perspectives on Phylogenetics and Biochemistry

Although the initial concept of chemotaxonomy in sponges could not fulfill its original expectations, which was resolving the complex classification of sponges, its continuous growing data source based on comprehensive records on metabolite distribution across all sponge classes complements other taxonomic methods. With rapid advancements and increasing versatility of molecular methods, modern sponge systematics substantially rely on the precision of complex genomic phylogenetic reconstruction models, the still present conflicts with phylogenies based on morphological characters notwithstanding, e.g., [178]. Detailed metabolite distribution patterns are a valuable asset in the resolution of such conflicting phylogenies, as the taxonomic allocation of “apomorphic” compounds often fits the topologies of molecular phylogenies well (see Figure 1). This genomically supported specificity of complex compounds furthermore makes convergent evolution of different metabolite groups increasingly unlikely.

Despite bacterial and fungal sources having taken the lead in reports of newly discovered marine natural products in the past few years, sponges remain the most prolific source of secondary metabolites and an important keystone in compound research [32]. Suitable and robust compound markers specific to sponge clades are, however, heavily reliant on the availability and reliability of information on these metabolites, hence causing a potential dynamic of applicability of markers over time (see Table 1). Substances prominently named after sponge species, like Latrunculin, Aaptamine, or Mycalolide, later on also being found in other (non-)Porifera clades, are just a few examples of mistakenly assumed exclusivity being revised on the basis of new findings, e.g., [164,210,211]. Although the overall number of “apomorphic” metabolite classes has increased since the reviews of van Soest and Braekman [45] and Erpenbeck and van Soest [46], many of the initial obstacles preventing correct metabolite allocation still persist in the present day. 

The most concerning problem, lacking or potential misidentifications, could be greatly alleviated by mandatory provision of DNA barcodes of frequently used marker regions (e.g., CO1, 28S, ITS) for studies on extraction and identification of novel marine natural products from sponges, in addition to detailed morphological descriptions and taxonomic identifications of the studied sponge specimens, conducted by experts on sponge taxonomy. As a consequence, compounds could be assigned to the correct species with more reliability and could quickly be checked for incongruences with morphological identifications. This would in turn also provide advantages for biochemical applications and metabolite screening, as more precise chemo-molecular phylogenies might provide further insights into the evolutionary pathways of metabolite classes and potentially promising taxa. This concept has however further room for improvement, as many biosynthetic pathways, involved genes, and the role of microbial symbionts are often not thoroughly understood yet, and might help to further comprehend the complex distribution patterns and evolution of secondary metabolites among sponge clades [212]. Investigations of the sponge microbiome have shown that microbial associations in sponges are to a large extent species specific [213,214,215]. Knowing the associated microbiome, potential function, and biosynthetic potential might help to identify if compounds are likely of microbial origin or produced by the sponge host [216]. This could be another approach for future studies to determine if compounds present in specific sponges could be used as phylogenetic markers.

Additional support in defining phylogenetic markers can be provided by metabolomic studies. The recent advances in nuclear magnetic resonance (NMR) technology and high-resolution mass spectrometry (HRMS) provides powerful resources for fast and exact structure determination of secondary metabolites. The increasing publication efforts on natural products by chemists and chemical ecologists have contributed to many different commercial databases like SciFinder (www.scifinder.cas.org, accessed on 29 July 2021), natural products libraries such as AntiBase (www.wiley-vch.de/stmdata/antibase.php, accessed on 29 July 2021) or Dictionary of Natural Products (dnp.chemnetbase.com, accessed on 29 July 2021). In addition, there are non-commercial, free of use databases such as ChemSpider (www.chemspider.com, accessed on 29 July 2021), PubChem (pubchem.ncbi.nlm.nih.gov, accessed on 29 July 2021), or Metlin (metlin.scripps.edu, accessed on 29 July 2021). Another approach is based on tandem mass spectrometry, where molecular ions are fragmented via MS/MS and resulting data analyzed via molecular networking. The crowdsourced Global Natural Products Social (GNPS) molecular networking website (http://gnps.ucsd.edu, accessed on 29 July 2021) is an open-access knowledge base. It enables natural product chemists to share their MS/MS spectrometry data for dereplication of known compounds and identification of potential new compounds [217,218,219,220]. These metabolomic approaches will surely accelerate compound assignment in sponges [221] and, combined with the latest DNA barcoding technology for sponge phylogeny, increase the list of natural product classes/compounds for phylogenetic markers in sponges.

## Figures and Tables

**Table 1:** Compilation of taxon specificity of investigated compound groups and comparison of the results in this study (as ‘2020′) with previous reviews in van Soest and Braekman [45] and Erpenbeck and van Soest [46], denoted by their respective year of publication. Plus sign = taxon specific; minus sign = nonspecific/unsuitable; circle = unresolved/conflicting information.

Metabolite Class	Taxonomic Group	1998	2004	2020
Pyrroloquinoline alkaloids	Poecilosclerida	O	+	+
Norditerpene peroxides	Podospongiidae (Poecilosclerida)	n.a.	n.a.	+
Norsesterterpene peroxides	Poecilosclerida	O	+	+
Pentacyclic guanidine alkaloids	*Monanchora* (Poecilosclerida)	+	+	+
Tetramic acids	Tetractinellida	n.a.	O	+
Steroidal saponins/glycosides	Tetractinellida	O	-	O
Isomalabaricane triterpenoids	Astrophorina (Tetractinellida)	+	+	+
Bengamide and bengazoles	Ancorinidae (Tetractinellida)	n.a.	O	+
Hydroxyiminosterols	*Cinachyrella* (Tetractinellida)	n.a.	+	+
Azetidine alkaloids	*Penares* (Tetractinellida)	+	+	+
3-Alkylpiperidines + 3-Alkylpyridines	Haplosclerida	+	-	+
Renieramycins	Haplosclerida	O	O	+
Straight-chain polyacetylenes	Haplosclerida	+	O	-
Pentacyclic hydroquinones	Petrosiidae (Haplosclerida)	n.a.	n.a.	+
3β-Hydroxymethyl-A-nor-sterols	Axinellida	n.a.	+	+
Cyanthiwigin diterpenes	*Myrmekioderma* (Axinellida)	O	+	+
Diterpene iso/thio/cyanides + formamides	Bubarida	O	O	+
Sesquiterpene iso/thio/cyanides + formamides	Bubarida	O	O	+
Carbonimidic dichlorides	Formerly Halichondrida	n.a.	O	-
Aaptamines	Suberitida	+	-	-
Suberitane-derived sesterterpenes	Suberitida	n.a.	+	-
Pyrrole-2-aminoimidazole alkaloids	Agelasida	+	+	+
Adenine-derivatives of diterpenes	* Agelas * (Agelasida)	n.a.	n.a.	+
Hypotaurocyamine (Sesquiterp. derivatives)	* Agelas * (Agelasida)	+	+	+
Bromotyrosines	Verongiida	+	-	+
Sesquiterpene lactones/furans	Dysideidae (Dictyoceratida)	O	-	+
Diterpene lactones/furans	Dendroceratida + Dictyoceratida	O	+	+
Sesterterpene lactones/furans	Spongiidae, Thorectidae, Irciniidae (Dictyoceratida)	O	-	+
Scalarane sesterterpenes	Spongiidae, Thorectidae, Irciniidae (Dictyoceratida)	n.a.	+	+
Scalarane sesterterpene hydroquinones	*Dysidea* + *Acanthodendrilla* (Dictyoceratida)	n.a.	n.a.	+
Polyprenylated benzoquinones	Irciniidae (Dictyoceratida)	n.a.	n.a.	+
Thiazole polyketides	Thorectidae (Dictyoceratida)	n.a.	n.a.	+
Polybrominated diphenyl ethers	Dysideidae (symbiotic origin) (Dictyoceratida)	n.a.	-	+
Cholest-5-en-3β-ol/5α(H)-cholestan-3β-ol	Hexactinellida	n.a.	+	+
Glycoceramides	Hexactinellida	n.a.	n.a.	+
Peroxy-Polyketides	*Plakortis* + *Plakinastrella* (Homoscleromorpha)	O	-	O
Steroidal alkaloids	*Plakina* + *Corticium* (Homoscleromorpha)	+	+	+
C_27_ to C_29_Δ^5,7,22^ & C_27_ to C29Δ^5,7,9(11),22^ sterols	Calcarea	n.a.	n.a.	-
Long-chain aminoalcohols	Clathrinida (Calcarea)	+	O	O
